# Starling Medical College

**Published:** 1848-12

**Authors:** 


*Starling Medical College.*—Lynde Starling, Esq., of Columbus, Ohio, has added to his donation of $30,000 to the Medical Institution bearing his name, an additional sum of $5000. A college edifice, including a small hospital with thirty beds, is to be commenced next spring. The generous donor deserves to be canonized in the calendar of medical saints, for we suspect such a munificent endowment of a medical College by a single individual, is without a precedent in this country.

